# Impact of *Aspergillus oryzae*-Derived Aminopeptidase Complex in Developing the Flavor Profile of Clam Hydrolysate

**DOI:** 10.3390/foods15101753

**Published:** 2026-05-15

**Authors:** Ting Zhao, Yibing He, Ying Han, Qinhao Liu, Xinqi Jian, Wei Zhao, Chiyue Zhang, Xianbing Xu, Yiying Nian, Zhenyu Wang, Ming Du, Peng Liu, Liming Sun

**Affiliations:** 1 SKL of Marine Food Processing & Safety Control, National Engineering Research Center of Seafood, School of Food Science and Technology, Dalian Polytechnic University, Dalian 116034, China; ztttin1729@163.com (T.Z.); hyb1730393764@163.com (Y.H.); hy17584566660@163.com (Y.H.); 16639738863@163.com (Q.L.); 18641785071@163.com (X.J.); 13352233260@163.com (W.Z.); 19741169626@163.com (C.Z.); xianbingxu@dlpu.edu.cn (X.X.); wangzhenyu@dlpu.edu.cn (Z.W.); duming@dlpu.edu.cn (M.D.); 2 Institute of Agriculture and Food Standardization, China National Institute of Standardization, Beijing 100191, China; nianyy@cnis.ac.cn

**Keywords:** *Aspergillus oryzae*, aminopeptidase–protease complex, clam, enzymatic hydrolysis

## Abstract

In our preliminary work, a clam sauce prepared by fermentation with *Aspergillus oryzae* 3.042 (AO) exhibited desirable flavor and quality; however, the process was prolonged (exceeding 30 d), and a high salt concentration (6–15%) was necessary to prevent spoilage. Consequently, shortening production cycle and reducing salt content without compromising product quality became a new objective. Enzymatic hydrolysis has long been recognized as an efficient approach in seasoning production, with enzyme efficacy being a key competitive factor. Accordingly, an AO-derived aminopeptidase–protease complex (AOAP) was optimized and prepared as a preparatory step. In this study, AOAP was applied to hydrolyze clam meat to evaluate its potential for producing a seasoning base. A two-step enzymatic hydrolysis process was employed. In the first step, the highest hydrolysis degree (29.1%) was achieved using alkaline protease (AP). The resulting hydrolysate was subsequently subjected to secondary hydrolysis with AOAP, achieving a degree of hydrolysis as high as 49.8%. Sensory evaluation revealed a significant reduction in bitterness and enhancement of umami in the final hydrolysate, a finding corroborated by electronic tongue analysis. Further characterization via LC-MS and amino acid (aa) analysis showed that a substantial number of bitter and umami peptides were released following AP treatment; however, the number of these peptides was markedly reduced after a subsequent AOAP hydrolysis, with concurrent substantial changes in the peptide profile. In the two-step hydrolysate, umami peptides mostly contain 3–10 aa, whereas bitter peptides typically contain only 3–5 aa. The content of free aa increased from 369.17 mg/100 g in the control to 3026.25 mg/100 g in the two-step hydrolysate, half of which were bitter, indicating the debittering efficiency of AOAP. Electronic nose analysis revealed similar flavor profile and characteristic presence of nitrogen oxides in all hydrolysates. GC-MS analysis further demonstrated that, after combined enzymatic hydrolysis, the short-chain aldehydes and ketones responsible for the fishy odor in the raw material almost completely disappeared, while long-chain aldehydes with pleasant aromas were generated. These findings suggest that the secondary hydrolysis step using AOAP can effectively improve the overall flavor profile of the clam hydrolysate, which may support its potential applicability in seasoning production, though further optimization and scale-up validation are needed.

## 1. Introduction

Condiments play an indispensable role in cooking, the food industry, nutritional health, and cultural heritage. Umami represents the most critical flavor component of traditional sauce-type condiments. The distinct and intense umami character of aquatic products constitutes a primary driver of consumer acceptance. Consequently, aquatic raw materials have long been established as essential resources for the production of natural flavoring condiments. In particular, seafood-derived condiments exhibiting superior umami profiles occupy a prominent position within the global condiment market.

Shellfish have long been favored by consumers owing to their desirable taste, tender texture, and affordability [1]. Beyond serving as a source of high-quality protein, shellfish meat is rich in nutrients, including monounsaturated and polyunsaturated fatty acids, as well as various trace elements [2,3]. While a significant proportion of shellfish is consumed fresh, the majority of processed products encompass frozen commodities and ready-to-eat snacks. In recent years, given the abundance of flavor-active substances present in shellfish [4], research initiatives and product development efforts aimed at utilizing shellfish as a base for seasoning or condiment production have garnered increasing attention [5,6].

Fermentation represents a traditional approach for the preparation of condiments. The utilization of fermented shellfish as a base for condiment production has been documented in previous studies [7]. It has been demonstrated that the amino acid composition undergoes substantial modification during fermentation, and the newly generated amino acids may contribute significantly to the taste and overall flavor profile of the fermented product [8,9,10]. However, such traditional fermentation processes typically require extended durations—often several months—to achieve a complex, mellow, and satisfactory flavor. Throughout this prolonged fermentation period, the addition of substantial quantities of salt is necessary to inhibit the proliferation of spoilage microorganisms [11]. Research indicates that proteolysis constitutes one of the principal biochemical events occurring during fermentation. The degradation products arising from this proteolytic activity, including amino acids and peptides, contribute substantially to various attributes of the final product, encompassing both nutritional quality and sensory properties [8]. Consequently, following the principles of fermentation, enzymatic hydrolysis has emerged as a prominent research focus over the past two decades. Investigators have employed commercial proteases to optimize hydrolysis processes, with the objective of attaining elevated degrees of hydrolysis or enhanced flavor characteristics [12,13,14,15]. Moreover, bioactive peptides have been identified as byproducts of these enzymatic treatments [16,17,18]. In recent years, novel auxiliary methodologies—such as peptidomics, virtual screening, and molecular docking—have been developed to facilitate the screening of umami peptides from hydrolysates and to elucidate their underlying mechanisms of action [19,20].

These investigations have demonstrated that enzymatic hydrolysis affords distinct advantages with respect to flavor development, nutritional quality, process efficiency, and controllability. Nevertheless, the flavor profiles of enzymatic hydrolysates prepared using the currently limited repertoire of commercial proteases are highly susceptible to pronounced homogenization, thereby impeding the development of distinctive seasoning or condiment products. Moreover, the quality of hydrolysates may exhibit considerable variability even when the same type of protease—albeit sourced from different manufacturers—is employed. Consequently, for a given protein substrate, the establishment of a stable protease production process is imperative to ensure the consistent generation of flavor peptides.

*Aspergillus oryzae* is a well-recognized and highly efficient protease-producing strain extensively employed in the manufacture of fermented foods [21]. As a class of exopeptidases, aminopeptidases possess the capacity to precisely and efficiently remove N-terminal hydrophobic amino acids, thereby exerting a debittering effect [22]. Aminopeptidases find widespread application in the modern food processing industry for a diverse array of purposes, including the debittering of protein hydrolysates [23,24,25], the enhancement of hydrolysate bioactivity [26], and the improvement of the degree of hydrolysis [27]. These functional attributes have prompted researchers to explore and identify novel aminopeptidases derived from *Aspergillus oryzae*, as well as to optimize the conditions governing their production [28,29].

Zhou et al. [30], from our laboratory, previously investigated the *Aspergillus oryzae*-mediated fermentation process on the volatile compound content and flavor characteristics of the resulting clam sauces. Their findings revealed that clam sauce prepared with soybean koji exhibited superior flavor and quality. This observation suggests that the hydrolysis of clam proteins and the subsequent development of flavor are primarily mediated by enzymes secreted by *Aspergillus oryzae* present in the soybean koji. Nevertheless, the fermentation of clam soy sauce necessitates a minimum duration of 30 days and entails a high salt concentration of 12%. Given that salt reduction is of considerable importance for human health, yet lowering the salt content substantially elevates the risk of fermentation failure attributable to contamination by spoilage microorganisms, alternative strategies are warranted. By contrast, the application of proteases derived from *Aspergillus oryzae* to hydrolyze clam meat not only obviates the requirement for high salt concentrations and the associated risk of spoilage but also substantially abbreviates the production cycle.

To explore the feasibility of rapid enzymatic preparation of a clam-flavored hydrolysate, an aminopeptidase with suitable catalytic activity is a key factor. The present study therefore focused on obtaining and evaluating an AOAP complex under laboratory conditions. In pursuit of this objective, we previously employed *Aspergillus oryzae* 3.042 to optimize the culture conditions and extraction procedures for aminopeptidase (AOAP) production via solid-state fermentation. The obtained AOAP complex was capable of hydrolyzing at least eight substrates (leucyl, phenylalanyl, glutamyl, methionyl, aspartyl, glycyl, alanyl, and prolyl-*p*-nitroanilines), exhibiting a novel enzyme activity profile, among which leucine aminopeptidase showed the strongest activity, followed by phenylalanine aminopeptidase, and then methionine aminopeptidase and glutamate aminopeptidase, while the activities of other types are weaker. Preliminary hydrolysis and sensory evaluation found that the hydrolysate obtained by the synergistic use of alkaline protease and AOAP had the best flavor and was close to that of fermented products. Therefore, the effect of AOAP on the hydrolysis of clam protein should be further investigated to confirm its potential application value in the preparation of clam condiment. In the present study, the fundamental enzymatic properties of AOAP were characterized, and the enzyme was subsequently applied in the preparation of a clam-flavored hydrolysate utilizing clam protein as the substrate. The primary emphasis was directed toward evaluating the effects of AOAP on debittering, the elimination of fishy off-odors, and the synergistic enhancement of umami taste in the resulting clam hydrolysate.

## 2. Materials and Methods

### 2.1. Materials

*Aspergillus oryzae* 3.042 was purchased from Shandong Hezhong Kangyuan Biotechnology Co., Ltd. (ZiBo, China). Frozen boiled clam (*Ruditapes philippinarum*) meat was supplied by Dandong Zhengrun Food Co., Ltd. (DanDong, China). Medium components, including wheat bran and corncob powder, were obtained from Beijing Hongrun Baoshun Technology Co., Ltd. (Beijing, China).

The following substrates were procured from Shanghai Aladdin Biochemical Technology Co., Ltd. (Shanghai, China): L-leucine-*p*-nitroaniline (Leu-pNA), L-alanine-4-nitroanilide (Ala-pNA), L-glycine-4-nitroanilide (Gly-pNA), L-glutamic acid-4-nitroanilide (Glu-pNA), L-phenylalanine-4-nitroanilide (Phe-pNA), L-methionine-4-nitroanilide (Met-pNA), L-aspartic acid-4-nitroanilide (Asp-pNA), L-proline-4-nitroanilide (Pro-pNA), and cyclohexanone. All aforementioned substrates were of analytical grade. Alkaline protease, acid protease, neutral protease, and other proteases (all of analytical grade) were obtained from Shanghai Yuanye Biotechnology Co., Ltd. (Shanghai, China). All additional chemicals and solvents employed in this study were of analytical grade.

### 2.2. AOAP Preparation

Spores of *Aspergillus oryzae* were inoculated at a final concentration of 10^8^ spores/mL into a medium containing 20% corncob powder as the sole nitrogen source. The initial pH of the medium was adjusted to 8.0, and the moisture content was maintained at 50%. The inoculated medium was subsequently incubated at 30 °C under a relative humidity of 70% for a duration of 60 h.

Following fermentation, deionized water was added to the solid-state fermentation medium at a solid-to-liquid ratio of 1:10 (g/mL). The resulting mixture was subjected to extraction in a constant-temperature water bath at 40 °C with continuous agitation for 1.5 h. The mixture was then subjected to preliminary filtration through two layers of gauze, and the filtrate was collected. The collected filtrate was centrifuged at 8000 rpm for 10 min, and the resulting supernatant was retained as the crude enzyme solution.

The crude enzyme solution was subsequently concentrated via fractional precipitation with ammonium sulfate. The target protein was precipitated within the 60–80% saturation range. The precipitate was harvested by centrifugation at 8000 rpm for 10 min, redissolved in deionized water, and desalted using a 20 kDa dialysis membrane. The desalted solution was further concentrated and purified by ultrafiltration using a 50 kDa molecular weight cut-off membrane. The purified enzyme solution thus obtained was designated as AOAP and stored at −20 °C pending further analysis.

### 2.3. AOAP Activity Determination

Total aminopeptidase activity in the sample was determined using L-leucine-*p*-nitroanilide (Leu-pNA) as the substrate and was expressed as Leu-aminopeptidase activity. The enzyme solution was diluted 10-fold with 50 mmol/L Tris–HCl buffer (pH 9.0). A portion of the diluted enzyme solution was inactivated by heating in a boiling water bath at 100 °C for 20 min to serve as the experimental control. Subsequently, equal volumes of the inactivated and non-inactivated enzyme solutions were each added to 1.2 mL of Tris–HCl buffer, and the resulting mixtures were pre-incubated at 50 °C for 10 min to ensure thermal equilibration. Thereafter, 100 μL of 25 mmol/L Leu-pNA solution was introduced into each sample tube, and the enzymatic reaction was allowed to proceed in a water bath at 50 °C for 10 min. The absorbance of the reaction mixture was subsequently measured at 405 nm. Aminopeptidase activity was calculated according to the following equation: where *K* represents the slope of the standard curve, *n* denotes the dilution factor, *A*_0_ corresponds to the absorbance of the blank control, and *t* signifies the reaction time. A series of *p*-nitroaniline solutions at concentrations of 10, 20, 30, 40, 60, and 80 μg/L were prepared using absolute ethanol as the blank control. The absorbance of each standard solution was measured at 405 nm, and the standard curve was generated by plotting the concentration of *p*-nitroaniline (μg/L) on the abscissa against the corresponding absorbance values on the ordinate.

(1) $$Aminopeptidase\;activity(U/mL)=\frac{A-A_{0}}{K}\times \frac{14n}{t}$$

### 2.4. Enzymatic Properties of AOAP

#### 2.4.1. Substrate Specificity

The substrate specificity of AOAP was analyzed using Leu-pNA, Ala-pNA, Gly-pNA, Glu-pNA, Phe-pNA, Met-pNA, Asp-pNA, and Pro-pNA as substrates, respectively, according to the method described in Section 2.3.

#### 2.4.2. Optimum Reaction Temperature and Thermal Stability

To determine the optimum reaction temperature of AOAP, the incubation temperature of 50 °C specified in Section 2.3 was replaced with 30, 40, 50, 60, and 70 °C, respectively. For the assessment of thermal stability, the AOAP solution was incubated at 0 (control), 20, 40, 60, and 80 °C for a duration of 1 h. Following incubation, all sample tubes were immediately placed in an ice bath for 5 min to terminate thermal effects, after which the residual aminopeptidase activity was measured in accordance with the procedure described in Section 2.3.

#### 2.4.3. Optimal Reaction pH and pH Stability

The optimal reaction pH of AOAP was determined by measuring enzyme activity in Tris–HCl buffers adjusted to pH values of 3.0, 5.0, 7.0, 9.0, and 11.0, following the method outlined in Section 2.3. For the evaluation of pH stability, the AOAP solution was incubated in the aforementioned buffers at the respective pH values and maintained in an ice bath at 0 °C for a period of 1 h. Subsequently, the residual aminopeptidase activity was assayed to determine pH stability.

### 2.5. Determination of Degree of Hydrolysis

The degree of hydrolysis (DH) of the hydrolysate was determined using the 2,4,6-trinitrobenzenesulfonic acid (TNBS) method [31]. Immediately following enzymatic hydrolysis, the hydrolysate was heated in a boiling water bath for 15 min to inactivate the enzyme. The mixture was subsequently centrifuged at 8000 rpm for 10 min, and the resulting supernatant was collected and diluted 600-fold. A 10 μL aliquot of the diluted supernatant was mixed with 200 μL of phosphate-buffered saline (PBS) buffer (pH 8.2) and 200 μL of 0.05% (*w*/*v*) TNBS aqueous solution. The reaction mixture was incubated at 50 °C for 1 h under dark conditions. The reaction was terminated by the addition of 400 μL of 0.1 mol/L HCl, and the absorbance was measured at 340 nm. An unhydrolyzed clam sample was subjected to the identical procedure and served as the blank control.

A standard curve was constructed using L-leucine as the standard to quantify the L-leucine content in the hydrolysate. The degree of hydrolysis (DH) was calculated using the following formula, where *C*_s_ represents the concentration of L-leucine in the clam hydrolysate, *C*_0_ = the L-leucine in blank control group, *C*_t_ = the L-leucine in the clam protein after complete hydrolysis.

(2) DH(%) = [(*C*_s_ − *C*_0_)/(*C*_t_ − *C*_0_)] × 100

Complete hydrolysis of clam protein was conducted according to the following procedure. Based on the protein content of the sample, 10–15 mL of 6 mol/L hydrochloric acid was added to the clam protein powder. The tubes were evacuated and flushed with nitrogen, and this process was repeated three times prior to sealing. The sealed tubes were subsequently incubated in a constant-temperature forced-air drying oven at 110 ± 1 °C for a duration of 22 h. Following hydrolysis, the tubes were cooled to room temperature, and the pH of the hydrolysate was adjusted to 7.0 by the addition of an equal volume of 6 mol/L NaOH.

### 2.6. Preparation of Clam Hydrolysate

#### 2.6.1. Pretreatment of Clam Meat

The viscera were removed from the clam meat. Deionized water was added at a ratio of 1:1 (*w*/*v*), and the mixture was homogenized at 3000 rpm for 1 min. The resulting homogenate was subsequently freeze-dried for a duration of 72 h until completely dry. The dried material thus obtained was ground into freeze-dried clam meat powder for subsequent use.

#### 2.6.2. First-Stage Enzymatic Hydrolysis

Each tube contained 2 g of clam meat powder, to which deionized water was added at a solid-to-liquid ratio of 1:50 (g/mL). Various commercial proteases and AOAP were individually added at an equivalent dosage of 5000 U/g protein, and hydrolysis was conducted under the respective optimal pH and temperature conditions for each enzyme for a period of 6 h. The supernatant of each hydrolysate was collected for the determination of the degree of hydrolysis (DH). Based on the DH values obtained, the protease yielding the highest DH was selected as the most suitable enzyme for the first-stage enzymatic hydrolysis of clam protein. The reaction conditions employed for each commercial protease are summarized in Table 1 according to the manufacturer’s recommended conditions. The final enzyme activity used was determined based on previous optimization experiments.

#### 2.6.3. Two-Stage Hydrolysis

AOAP freeze-dried powder was added to the hydrolysate obtained from the first-stage hydrolysis at a dosage of 5000 U/g protein. The pH was adjusted to 7.0 instead of the enzyme’s optimal pH of 9.0, because AOAP exhibited relatively poor operational stability at pH 9.0. Therefore, a neutral pH (7.0) was selected to maintain enzymatic activity during the extended hydrolysis reaction. The hydrolysis was performed at 50 °C for 0, 1, 2, 3, 4, 5, and 6 h, respectively. Following each time point, the mixture was centrifuged at 8000 rpm for 10 min, and the resulting supernatant was collected. The optimal hydrolysis time was determined by analyzing the changes in DH over the time course.

Based on the above procedure, four groups were set up: clam protein alone (control), clam protein hydrolyzed with alkaline protease (alkaline protease group), clam protein hydrolyzed with AOAP (AOAP group), and clam protein hydrolyzed sequentially with alkaline protease and then AOAP (combined hydrolysis group, alkaline protease + AOAP).

### 2.7. Sensory Evaluation

The sensory characteristics of the clam hydrolysate were evaluated using quantitative descriptive analysis combined with a numerical scoring method. The samples were presented in 20 mL sample cups and assessed by a panel of 12 trained sensory assessors (six males and six females), aged 20 to 25 years. Panelists were selected from healthy non-smoking research personnel after passing a basic taste recognition test (accuracy ≥80%). They then completed three training sessions using L-glutamate monosodium salt as an umami reference and caffeine as a bitterness reference to standardize scoring. Panelists rinsed their mouths with purified water between samples. The samples were presented in a randomized order, and the panelists were blinded to sample identity using three-digit random codes. Each sample was evaluated in three independent replicates, and the mean score from the three replicates was calculated for each attribute. A 0–10 scoring scale was employed to rate the intensity of the following attributes: aroma, umami, fishy odor, bitterness, color, and overall acceptability. Higher scores corresponded to a greater perceived intensity of the respective attribute. The final score for each attribute was calculated as the mean value derived from the individual ratings provided by the panelists. Sensory evaluation was conducted in a dedicated sensory analysis laboratory under controlled ambient conditions. No communication was permitted among the panelists during the evaluation, and the ambient temperature was maintained at 25 °C. The detailed scoring criteria employed in the sensory evaluation are presented in Table 2.

### 2.8. Taste Profile Analysis by Electronic Tongue

The taste profiles of the clam hydrolysates were analyzed using a TS-5000Z electronic tongue system from Insent Inc. (Tokyo, Japan). Prior to analysis, all samples were filtered through a 0.22 μm membrane filter. The sensor responses were calibrated using a standard reference solution (30 mM KCl + 0.3 mM tartaric acid). Each sample was subjected to four replicate measurements using the following five taste sensors: C00 (bitterness), AE1 (astringency), CA0 (sourness), CT0 (saltiness), and AAE (umami). The data obtained from the first measurement cycle were discarded to ensure sensor stabilization, and the mean value of the subsequent three replicate measurements was calculated and reported as the final result.

### 2.9. Flavor Profile Analysis by Electronic Nose

The flavor profiles of the clam hydrolysates were analyzed using a PEN3 electronic nose system (WinMuster Airsense Analytics Inc., Schwerin, Germany). Prior to analysis, the system was preheated for 60 min to reach a stable operating temperature. The sensors were calibrated using zero gas (clean air filtered through activated carbon), and the baseline sensor response (G0) was defined as the conductance of the sensors in the zero gas. Sensor responses to samples were expressed as the conductance ratio (G/G0), where G is the conductance of the sensor upon exposure to the sample headspace. A total of 5 mL of each hydrolysate sample was transferred into a 20 mL headspace vial and sealed with Parafilm. Following the purging of the sensor probe with filtered air, the probe was inserted into the headspace of the vial, and data acquisition was conducted for a duration of 100 s. After each measurement, the sensor system was flushed with zero gas for 70 s to restore the baseline before the next sample analysis. The substances to which each type of sensing element is sensitive are listed in Table 3.

**Table 3:** Sensor array of the electronic nose.

MOS	General Description	Sensitive Gas
W1C	Aromatic compounds	Toluene, 10 ppm
W5S	Very sensitive to nitrogen oxides	NO_2_, 1 ppm
W3C	Ammonia, used as a sensor for aromatic compounds	Benzene, 10 ppm
W6S	Mainly hydrogen, selectively (breath gases)	H_2_, 100 ppb
W5C	Alkenes, aromatic compounds, less polar compounds	Propane, 1 ppm
W1S	Sensitive to methane broad range	CH_3_, 100 ppm
W1W	Reacts to sulphur compounds	H_2_S, 1 ppm
W2S	Detects alcohols, partially aromatic compounds	CO, 100 ppm
W2W	Aromatics compounds, sulphur organic compounds	H_2_S, 1 ppm
W3S	Reacts to high concentrations	CH_3_, 100 ppm

### 2.10. Peptide Composition Analysis by LC–MS/MS

The sample was desalted using a C18 column and subsequently analyzed by liquid chromatography–tandem mass spectrometry (LC–MS/MS) equipped with an online nano-electrospray ionization source. The system consisted of an EASY-nanoLC 1200 from Thermo Fisher Scientific coupled in tandem with an Orbitrap Fusion Lumos mass spectrometer (Waltham, MA, USA). A sample volume of 5 μL was injected onto an Acclaim PepMap C18 analytical column (75 μm × 25 cm). Separation was performed using a 60-min gradient. The column flow rate was maintained at 300 nL/min, the column temperature was held at 40 °C, and the electrospray voltage was set to 2 kV. The gradient was initiated at 4% mobile phase B, increased nonlinearly to 50% over a period of 53 min and 40 s, ramped to 95% within 40 s, and subsequently maintained at 95% for an additional 5 min and 40 s.

The mass spectrometer was operated in data-dependent acquisition (DDA) mode, with automatic switching between full MS and MS/MS scans. The mass spectrometric parameters were configured as follows: (1) Full MS: scan range (*m*/*z*) 100–1500; resolution 120,000; normalized AGC target 200%; maximum injection time 100 ms. (2) HCD-MS/MS: resolution 50,000; normalized AGC target 200%; maximum injection time 86 ms; collision energies 25%, 30%, and 35%; dynamic exclusion duration 30 s.

Chromatographic baseline was defined using the average background noise of blank retention time regions adjacent to each peptide peak, and baseline correction was performed prior to peak integration. Relative peptide quantification was carried out using baseline-corrected extracted ion chromatogram peak areas. Total ion current (TIC) normalization was used to reduce systematic variations from sample loading, instrumental drift and matrix effects, and normalized peak areas were adopted for subsequent group comparisons.

The prediction of umami and bitter peptides was conducted at the website http://umami.peptideinnov.com, and the graphs were plotted at https://www.chiplot.online/.

### 2.11. Determination of Amino Acid Composition

Clam hydrolysates were diluted 200-fold with deionized water. A 1 mL aliquot of the diluted sample was mixed with acetone at a ratio of 1:3 (*v*/*v*) and allowed to stand for 30 min to achieve complete protein precipitation. The mixture was subsequently centrifuged at 10,000× *g* for 5 min, and the resulting supernatant was collected. The supernatant was evaporated to dryness using a rotary evaporator, and the residue was reconstituted in 0.02 mol/L hydrochloric acid. The resulting solution was filtered through a 0.22 μm membrane filter and transferred into a 1 mL injection vial for analysis. Free amino acids were determined using a Hitachi LA8080 amino acid analyzer (Tokyo, Japan). Gradient elution was performed at a column temperature of 57 °C, and the injection volume was 20 μL.

### 2.12. Detection of Volatile Flavor Compounds

Volatile flavor compounds in the clam hydrolysates were analyzed by gas chromatography–mass spectrometry (GC–MS) using an Agilent 8890/7000E GC–MS system (Santa Clara, CA, USA) [32]. A 3 mL aliquot of each sample was transferred into a sampling vial. Volatile compounds were extracted by incubating the vial in the GC–MS heating module at 60 °C for 30 min with continuous agitation, followed by thermal desorption at 220 °C for 4 min. The extracted compounds were subsequently separated and detected using a 7890B gas chromatography system. The GC conditions were as follows: separation was achieved on an Agilent HP-5MS polar capillary column (30 m × 250 µm × 0.25 µm). The oven temperature was initially held at 40 °C for 2 min, then increased at a rate of 8 °C/min to 250 °C and maintained at this temperature for 10 min. The injector temperature was set at 250 °C. High-purity helium was employed as the carrier gas at a constant flow rate of 1.2 mL/min under splitless injection mode. The MS conditions were configured as follows: EI ion source; electron energy 70 eV; transfer line temperature 250 °C; ion source temperature 230 °C; quadrupole temperature 150 °C; and mass scan range *m*/*z* 45–450. Quantitative analysis of volatile flavor compounds was performed using the internal standard method.

### 2.13. Statistical Analysis

The experiment was conducted in three batches, and each measurement was repeated three times. The results are expressed as the mean ± standard deviation (SD) derived from three independent replicates. Analysis of variance (ANOVA) was performed using SPSS 23.0 statistical software (SPSS Inc., Chicago, IL, USA) to evaluate significant differences among samples. Post-hoc comparisons were conducted using Tukey’s multiple range test. Differences with a *p*-value < 0.05 were considered statistically significant. Data plotting was conducted using Origin Pro 9.0, and principal component analysis (PCA) was performed using MetaboAnalyst 5.0.3.

## 3. Results and Discussion

### 3.1. Enzymatic Properties of AOAP

AOAP exhibited hydrolytic activity toward several amino acid residues, and the substrate specificity of AOAP was determined to be in the following order: Leu > Phe > Glu = Met > Asp > Gly > Ala. The maximum enzymatic activity was observed at 50 °C and pH 9.0. The enzyme was relatively stable at temperatures below 60 °C and at pH values below 7.0; however, it was susceptible to inactivation under acidic conditions and in alkaline environments with pH values exceeding 9.0. Among the protease inhibitors tested, 1,10-phenanthroline exerted the most pronounced inhibitory effect on AOAP activity, indicating that AOAP belongs to the class of metalloproteinases. The activity of AOAP was significantly enhanced by the presence of Co^2+^ at concentrations ranging from 0.1 to 1 mM, whereas it was markedly inhibited by Ba^2+^ (see Supplementary Data).

### 3.2. Enzymatic Hydrolysis of Clams

**Figure 1:**
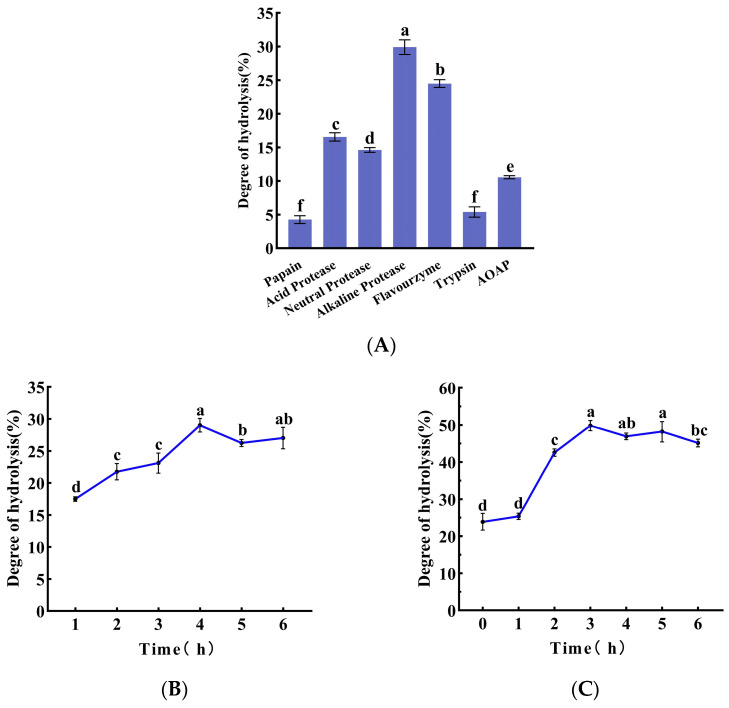
Changes in the degree of hydrolysis (DH) during enzymatic hydrolysis of clam protein. (**A**) Effect of AOAP and various commercial proteases on the DH of clam protein. (**B**) Time course of DH during hydrolysis with alkaline protease. (**C**) Second-stage hydrolysis process mediated by AOAP, initiated from the 4 h hydrolysate obtained with alkaline protease. Different lowercase letters indicate significant differences in the average value within each group (*p* < 0.05).

The effects of AOAP and various commercial proteases on the degree of hydrolysis (DH) of clam protein were compared, as presented in Figure 1A. The results demonstrate that following 6 h of hydrolysis with AOAP alone, the DH reached only 10.5%. This finding is consistent with the characteristic behavior of aminopeptidases as exopeptidases, which catalyze the sequential cleavage of amino acids from the N-terminus of peptide chains and are generally ineffective in directly degrading intact proteins. Consequently, AOAP should be employed in combination with an endo-protease. Among the commercial proteases evaluated, alkaline protease exhibited the highest hydrolytic capacity, achieving a DH of 29.1%, which was significantly superior to that obtained with the other proteases tested. Accordingly, alkaline protease was selected for the first-stage hydrolysis of clam protein. An investigation of the effect of hydrolysis time on the efficiency of alkaline protease revealed that the maximum DH was attained at 4 h (Figure 1B). The hydrolysate obtained under these conditions was subsequently subjected to a second-stage hydrolysis with AOAP, and the corresponding changes in DH are illustrated in Figure 1C. An enhanced degree of hydrolysis, from 23.9% to 49.8%, was achieved following 3 h of hydrolysis with AOAP.

Among the various commercial proteases, alkaline protease is an alkaline bacterial endopeptidase that has long been recognized as one of the most effective enzymes for the preparation of protein hydrolysates with respect to DH. However, a common drawback associated with its application is the pronounced bitterness exhibited by the resulting hydrolysate. Therefore, a subsequent debittering treatment with an aminopeptidase is generally required. Concomitantly, the flavor profile and composition of the hydrolysate undergo substantial modifications. The synergistic combination of alkaline protease and aminopeptidase has thus become a widely adopted strategy for the preparation of protein hydrolysates. Combined treatments with alkaline protease (endopeptidase) and aminopeptidase (exopeptidase) consistently achieved high protein yields and enhanced degrees of hydrolysis, producing lower-molecular-weight peptides [23,33]. The alkaline protease first hydrolyzes the peptide bonds at the carboxyl side of hydrophobic amino acids (Ala, Leu, Val, Phe) within the protein molecule, cleaving the intact protein into numerous medium- and long-chain peptides. This exposes more N-termini and peptide bond sites for the subsequent action of the aminopeptidase (an exopeptidase). The aminopeptidase then continuously removes N-terminal amino acids, which reduces product inhibition and shifts the hydrolysis equilibrium toward the forward reaction, thereby allowing the degree of hydrolysis to surpass the upper limit achievable by single-enzyme hydrolysis. Results presented in Figure 1C further corroborate this synergistic effect.

For the purpose of subsequent comparative analyses, four hydrolysate samples were designated as follows: the blank group (freeze-dried clam protein), the alkaline protease group (hydrolysate obtained following hydrolysis with alkaline protease for 4 h), the AOAP group (hydrolysate obtained following hydrolysis with AOAP for 4 h), and the two-stage hydrolysis group (hydrolysate derived from the alkaline protease group subjected to secondary hydrolysis with AOAP for an additional 3 h).

### 3.3. Sensory Evaluation of Clam Hydrolysates

**Figure 2:**
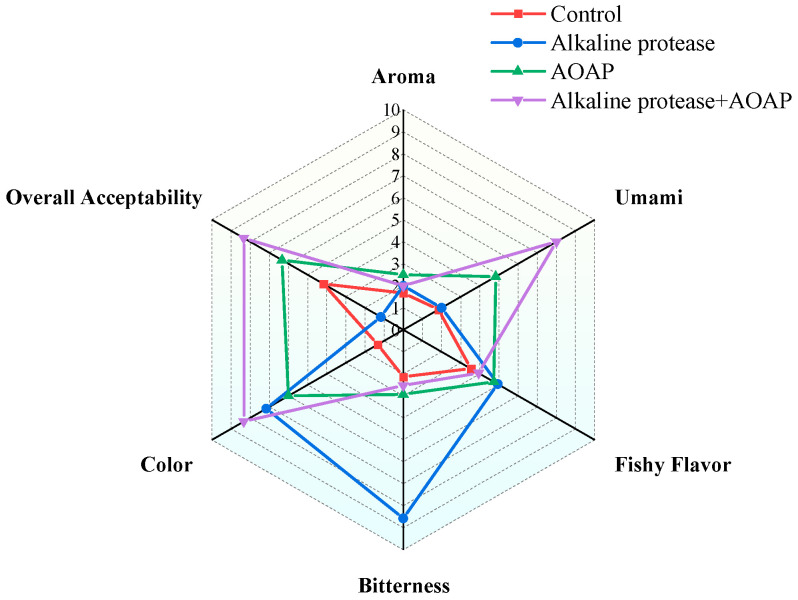
Sensory evaluation scores of the clam hydrolysates.

With respect to volatile flavor attributes, as illustrated in Figure 2, all four groups of hydrolysates exhibited weak aroma and moderate fishy odor intensities, with broadly similar flavor profiles observed across the groups. The most pronounced differences resulting from enzymatic hydrolysis were observed in the intensities of bitterness and umami. Following hydrolysis of clam protein with alkaline protease, bitterness increased substantially. However, upon subsequent treatment with AOAP, the perceived bitterness decreased significantly. Correspondingly, the umami intensity declined markedly after hydrolysis with alkaline protease, whereas it increased significantly following the secondary enzymatic hydrolysis with AOAP. Overall, the order of acceptability for the hydrolysates was as follows: combined enzymatic hydrolysis group > AOAP group > control group > alkaline protease group. These findings indicate that AOAP, whether applied alone or in combination with an endo-protease, is capable of enhancing the umami taste of clam hydrolysates, with a more pronounced enhancement observed under the combined enzymatic treatment. Future work needs to further optimize AOAP and the enzymatic hydrolysis conditions, conduct more detailed preference tests and preference ranking of the hydrolysates, and validate the results using bitter amino acids (e.g., Leu) and umami amino acids (e.g., Glu), so as to provide further constructive support for the practical application of this enzyme and product development.

### 3.4. Taste Characteristics of Clam Hydrolysates

**Figure 3:**
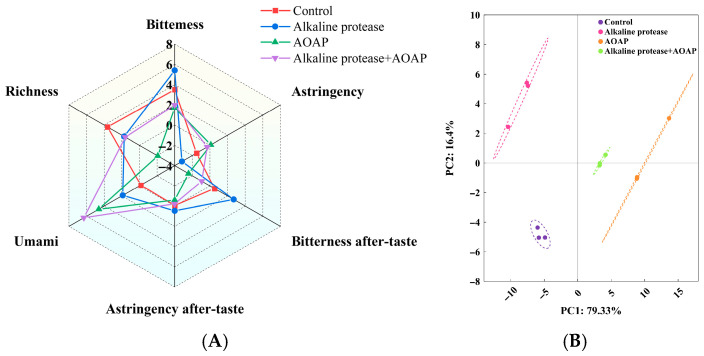
Electronic tongue analysis of clam hydrolysates. (**A**) Radar chart of taste attributes. (**B**) Principal component analysis (PCA) score plot.

The effects of enzymatic hydrolysis on taste attributes were evaluated using an electronic tongue, based on six indicators, including umami, bitterness, astringency, bitterness aftertaste, astringency aftertaste, and richness. As presented in Figure 3, enzymatic hydrolysis exerted a relatively weak effect on astringency and its associated aftertaste, whereas it exerted a pronounced effect on the remaining four indicators. AOAP, whether applied alone or in combination with an endo-protease, effectively reduced the bitterness and enhanced the umami intensity of the hydrolysate. Among the four groups evaluated, the most substantial differences were observed in umami and bitterness aftertaste. Specifically, the umami intensity increased in the following order: control group < alkaline protease group < AOAP group < combined enzymatic hydrolysis group. Concurrently, the bitterness aftertaste decreased in the following order: alkaline protease group > control group > combined enzymatic hydrolysis group > AOAP group. The observed differences in umami and bitterness aftertaste likely accounted for the clear separation of the four sample groups in the principal component analysis (PCA) results. Collectively, the findings presented in Figure 3 further corroborate the sensory evaluation results (Figure 2), thereby confirming the debittering and umami-enhancing effects of AOAP.

### 3.5. Flavor Profile Analysis of Clam Hydrolysates

**Figure 4:**
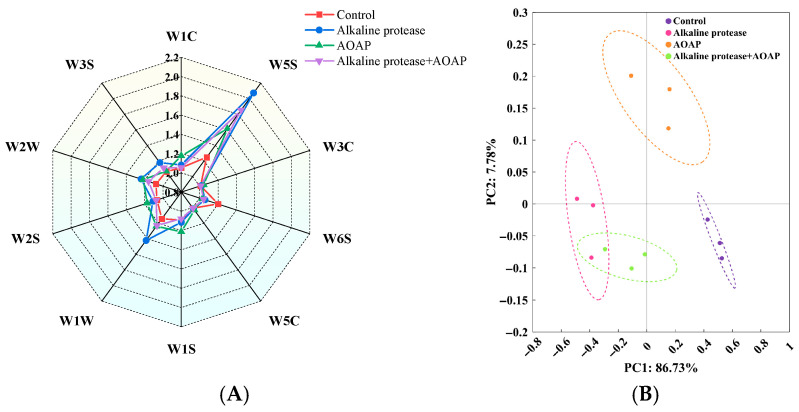
Electronic nose response profiles of clam hydrolysates. (**A**) Radar chart of sensor responses. (**B**) Principal component analysis (PCA) score plot.

The radar chart depicting the responses of the ten sensors of the electronic nose to the volatile flavor compounds of the clam hydrolysates is presented in Figure 4. Following hydrolysis of clam protein with alkaline protease, the response values of the W5S and W1W sensors increased significantly, whereas the response of the W6S sensor exhibited a slight decrease. These observations suggest that hydrolysis with alkaline protease led to an increase in the abundance of nitrogen oxides and sulfides, accompanied by a dehydrogenation reaction (i.e., an oxidation reaction). Overall, the most pronounced difference among the four hydrolysate groups was primarily reflected in the content of nitrogen oxides. This finding is largely consistent with the flavor profile of fermented clam sauce reported by Zhou et al. [30], indicating that the flavor characteristics of the enzymatic hydrolysate closely resemble those of the fermented product. The free amino acids generated during enzymatic hydrolysis serve as potential substrates for the formation of nitrogen oxides. The alkaline environment itself is capable of accelerating reactions such as deamination, decarboxylation, and the oxidation of amino acids, with the resulting intermediates being subsequently oxidized to nitrogen oxides [34]. Furthermore, both AOAP and alkaline protease are complex multi-enzyme systems derived from microorganisms, which may harbor nitric oxide synthase activity, thereby enabling the production of nitrogen oxides such as nitric oxide [35].

### 3.6. Peptide Composition of Clam Hydrolysates

#### 3.6.1. Peptide Species and Length Distribution

**Figure 5:**
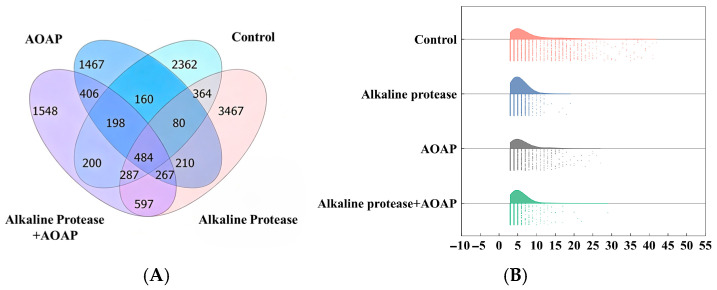
Peptide composition analysis of clam hydrolysates by LC–MS/MS. (**A**) Venn diagram illustrating the distribution of peptide species among the four hydrolysate groups. (**B**) Raincloud plot depicting the distribution of peptide lengths across the four hydrolysate groups.

In the present study, peptidomic analysis of the four groups of clam hydrolysates was conducted using nano-HPLC–MS/MS, and a total of 6337 peptides were identified. As presented in Figure 5A, a total of 2545, 3025, 1873, and 2252 peptides were detected in the blank group, alkaline protease group, AOAP group, and combined enzymatic hydrolysis group, respectively. Further analysis of the peptide length distribution (Figure 5B) revealed that the peptides in all groups were predominantly composed of 3–8 amino acid residues. Among all the detected peptides, the blank group contained a substantially higher number of large peptides (>10 amino acid residues) than the other groups. Following treatment with alkaline protease, the variety of large peptides was markedly reduced, accompanied by a corresponding increase in the abundance of small peptides. AOAP, when applied alone, did not significantly increase the number of small peptides. However, when employed in combination with alkaline protease, nearly all of the detected peptides consisted of 3–8 amino acid residues. This finding further confirms the exopeptidase nature of AOAP and underscores its considerable value in secondary hydrolysis. During AOAP-mediated secondary enzymatic hydrolysis, the molecular weight of the resulting products is further reduced and becomes more uniform, which is conducive to both flavor development and intestinal absorption [36,37].

#### 3.6.2. Composition Analysis of Umami and Bitter Peptides

**Figure 6:**
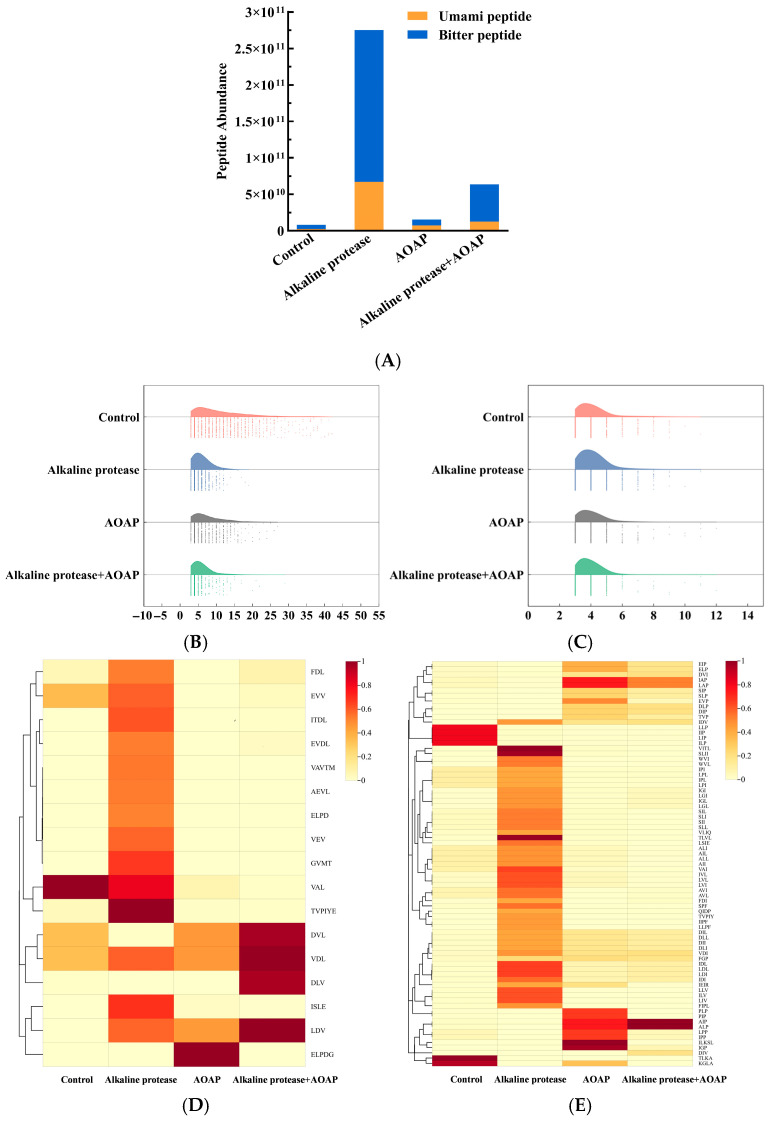
Predicted umami and bitter peptide composition of clam hydrolysates. (**A**) Stacked abundance plot of predicted umami and bitter peptides. (**B**) Raincloud plot depicting the length distribution of predicted umami peptides. (**C**) Raincloud plot depicting the length distribution of predicted bitter peptides. (**D**) Heatmap illustrating the relative abundance of predicted umami peptides. (**E**) Heatmap illustrating the relative abundance of predicted bitter peptides.

Umami-Transformer is a deep learning framework based on the Transformer architecture integrated with eight physicochemical descriptors, which was developed to achieve high-precision prediction of umami peptides through sequence encoding and feature fusion [38]. In the present study, the Umami-Transformer model was employed to predict umami peptides in the clam hydrolysates. As presented in Figure 6A, the contents of both in silico-predicted umami peptides and in silico-predicted bitter peptides in the blank group were extremely low. After treatment with alkaline protease, a large number of predicted bitter and umami peptides were released, whereas treatment with AOAP alone exerted a negligible effect. The alkaline protease group contained a substantial abundance of predicted bitter peptides; however, subsequent treatment with AOAP reduced the predicted bitter peptide relative abundance by 75.1%. A total of 3131 in silico-predicted umami peptides and 3206 in silico-predicted bitter peptides were identified across the four hydrolysate groups. The predicted umami peptides were predominantly 3–10 amino acid residues in length, whereas the predicted bitter peptides were primarily 3–5 residues in length (Figure 6B,C). Further heatmap analysis (Figure 6D,E) revealed that the unique peptides present in the alkaline protease group predominantly contained hydrophobic amino acids, such as leucine, isoleucine, valine, serine and threonine, at their N-termini. Aminopeptidases achieve debittering by specifically removing hydrophobic amino acids exposed at the N-termini of bitter peptides. The addition of AOAP markedly decreased the relative abundance of these peptides [22], further supporting its potential role in debittering.

Fu et al. [39] reported that peptides with a length of 9–10 amino acid residues are more likely to adopt favorable conformations and achieve low-energy binding to taste receptors, thereby exhibiting lower umami taste thresholds. Moreover, it has been documented that the bitterness of short peptides (2–5 amino acid residues) exhibits a terminal effect, whereby hydrophobic residues located at the C- and N-termini can increase bitterness intensity by 3- to 5-fold. Based on these previous findings (which were derived from experimentally validated peptides), combined with the observation that the majority of in silico-predicted umami peptides in the present study fell within the 3–10 residue range and the predicted bitter peptides within the 2–5 residue range, it is plausible to hypothesize that the length distribution of these predicted peptides may contribute to the reduced bitterness and enhanced umami taste observed in the two-stage enzymatic hydrolysate. AOAP may also exert its effects through two potential mechanisms: It cleaves amino acids from the precursor peptides generated by alkaline protease and reassembles or exposes peptide sequences exhibiting umami characteristics. It removes bitter peptides, enabling the umami peptides originally masked by bitterness to bind more effectively with taste receptors.

### 3.7. Amino Acid Composition Analysis of Clam Hydrolysates

**Figure 7:**
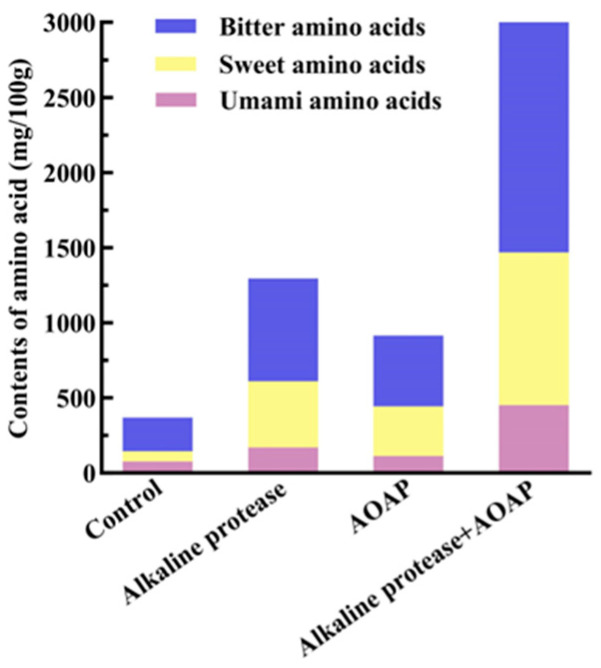
Stacked bar plot of free amino acid contents in clam hydrolysates.

As presented in Table 4 and Figure 7, in the control group, only four types of free amino acids (aa) were detected with very low content. Both the variety and the content of aa increased markedly following treatment with either alkaline protease (AP) or AOAP, whereas AP-AOAP hydrolysis yielded the highest levels, thereby demonstrating the efficiency of the synergistic hydrolysis approach. Compared with the AP hydrolysate, the contents of umami, sweet, and bitter aa in the combined enzymatic hydrolysate increased by 2.67, 2.30, and 2.28 folds, respectively. As fundamental flavor components, free aa can impart various taste attributes, including umami, sweetness, bitterness, and sourness [40]. It is well established that bitter peptides contribute more substantially to overall bitterness than do hydrophobic free aa, owing to the higher taste threshold of bitter aa relative to that of bitter peptides [41]. The abundant bitter aa detected in the final hydrolysate are precisely those released from bitter peptides through hydrolysis, thereby substantially reducing the bitterness of the hydrolysate derived from alkaline protease treatment.

Taste activity values (TAVs) were calculated, as presented in Table 5. Glutamic acid exhibited the highest TAV of 10.71, indicating that it served as the principal contributor to the umami taste of the hydrolysates. The sweet-tasting aa primarily included threonine, glycine, proline, and lysine, among which lysine displayed the highest TAV of 3.38. Sweet-tasting amino acids may interact synergistically with umami components via the T1R1/T1R3 taste receptor, thereby enhancing the overall perception of umami [42].

### 3.8. Volatile Flavor Compounds in Clam Hydrolysates

**Figure 8:**
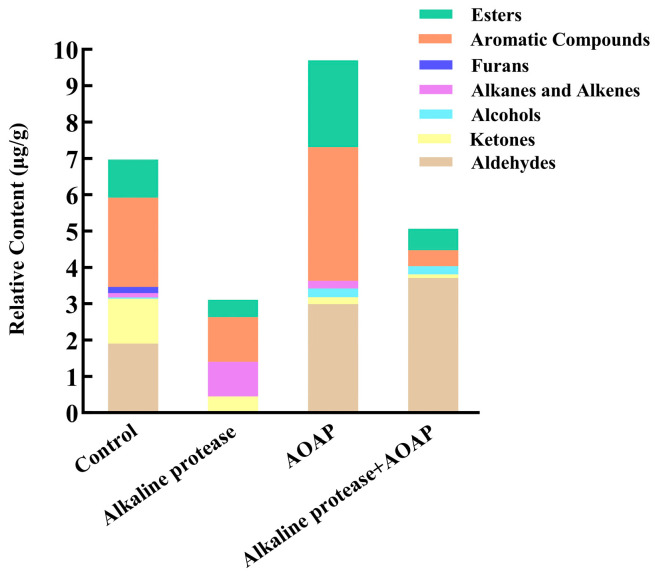
Stacked bar plot of the relative contents of volatile compounds in clam hydrolysates.

**Figure 9:**
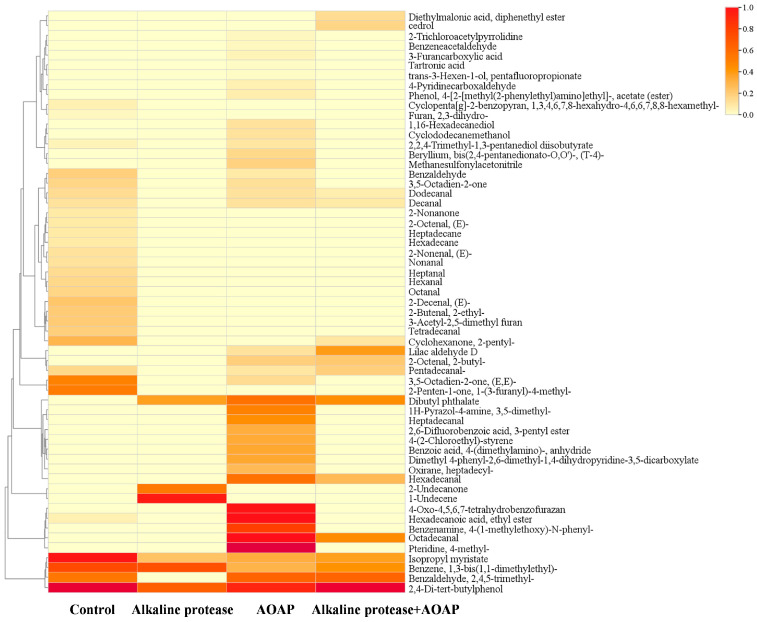
Heatmap illustrating the relative abundance of volatile compounds in clam hydrolysates.

The volatile components in the four clam hydrolysates were analyzed using HS–GC–MS. A total of 50 flavor compounds were identified, comprising 21 aldehydes, 6 ketones, 4 alcohols, 4 alkanes/alkenes, 8 esters, 5 aromatic compounds, and 2 furans. The changes in the profiles of the aforementioned compounds during hydrolysis are illustrated in Figure 8 and Figure 9. The relative contents of these compounds are summarized in Table 6.

Following treatment with alkaline protease, the total content of volatile compounds decreased significantly, and aldehydes were no longer detectable. The specific effect exerted by alkaline protease on clam protein warrants further investigation. In contrast, abundant aldehydes predominated in the control group and other hydrolysates, particularly in the combined enzymatic hydrolysate. Aldehydes represent the most typical flavor compounds in fresh or cooked seafood, owing to their low odor threshold values [14]. These compounds exhibit a pronounced synergistic effect, such that even when present in trace amounts, they contribute substantially to the overall flavor profile [43]. Among the aldehydes, short-chain species—including 2-ethylbutenal, trans-2-decenal, benzaldehyde, decanal, heptanal, hexanal, nonanal, and octanal—constitute the primary source of the fishy odor associated with clam protein (the control group) [44,45]. These short-chain aldehydes were nearly absent following combined enzymatic hydrolysis. Conversely, the contents of certain long-chain aldehydes, including dodecanal, hexadecanal, heptadecanal, and octadecanal, increased markedly in the combined hydrolysate. Several long-chain aldehydes have been reported to impart floral and sweet aroma notes [46]. In general, however, long-chain aldehydes, owing to their elevated boiling points, contribute minimally to the immediate aroma perception but serve as important precursors of flavor-active substances [47]. The content of 2,4,5-trimethylbenzaldehyde (characterized by a sweet, floral odor) remained at a relatively high and stable level across treatments. Notably, in the *Aspergillus oryzae*-fermented clam sauce prepared by Zhou et al. [30], the majority of detected aldehydes were short-chain species, with long-chain aldehydes being virtually absent.

With the exception of 2-pentylcyclohexan-1-one detected in the control group, all identified ketones have been associated with pungent and fishy odor notes [48]. Following enzymatic hydrolysis, the levels of these ketones decreased substantially, with several becoming undetectable. The principal aromatic compounds, namely 2,4-di-tert-butylphenol (characterized by a distinct alkylphenol-like odor) and 1,3-di-tert-butylbenzene (exhibiting a pungent odor), were present at substantial levels in the clam protein and in the hydrolysates derived from treatment with alkaline protease and AOAP; however, their contents declined significantly following combined enzymatic hydrolysis. Furan compounds, such as 3-acetyl-2,5-dimethylfuran (which contributes sweet, musty, and earthy notes), were detected exclusively in the blank group. Isopropyl myristate constituted the most abundant ester in the control group and decreased following hydrolysis; however, this ester is generally described as odorless or nearly odorless. Of particular note, dibutyl phthalate—a recognized marine pollutant—was detected in all hydrolysate samples, and its content remained relatively stable across treatments. But this pollutant was not detected in the fermented product prepared by Zhou et al. [30], a finding that further underscores the high efficiency of enzymatic hydrolysis in degrading substrate macromolecules. The liberation of this pollutant from the hydrolysate matrix facilitates its subsequent removal via techniques such as adsorption and biodegradation, thereby mitigating potential risks to human health. This observation points to a potential advantage of enzymatic hydrolysis over traditional fermentation, in that the release of certain pollutants may facilitate their subsequent removal; however, the efficiency of such removal and the overall risk reduction remain to be demonstrated.

Aldehydes, ketones, and aromatic compounds are the principal products of fatty acid degradation [49], arising primarily from the oxidative decomposition of unsaturated fatty acids such as oleic acid, linoleic acid, and arachidonic acid. These observations suggest that for aquatic products such as clams, which are characterized by a high content of unsaturated fatty acids, it is advisable to eliminate lipase and lipoxygenase activities, as well as other factors that promote lipid oxidation, from the enzyme preparation employed during enzymatic hydrolysis. Furthermore, degreasing of the raw material is recommended. Such measures would not only assist in controlling fatty acid oxidation within the hydrolysate but also, from a long-term perspective, extend the shelf life of the final product. Nevertheless, the potential impact of these interventions on product flavor warrants careful consideration.

## 4. Conclusions

As preliminary work in our laboratory, a clam sauce prepared by fermentation with *Aspergillus oryzae* 3.042 (AO) exhibited desirable flavor and quality. To reduce salt addition and shorten the processing time, we obtained an aminopeptidase complex (AOAP) from AO. This enzyme is alkaline, metal-dependent, and has broad substrate specificity. In this study, the effect of AOAP on the proteolytic hydrolysate of clam meat was investigated to explore whether enzymatic hydrolysis involving AOAP could replace the high-salt, long-term fermentation process. Compared with other commercial proteases, AOAP alone exhibited very low hydrolysis efficiency (10.55%), far lower than that of alkaline protease, which showed the highest efficiency (29.1%). However, when AOAP was used in synergy with alkaline protease, AOAP demonstrated a significant pro-hydrolysis effect, increasing the degree of hydrolysis to 49.8%. After treatment with alkaline protease, a large number of bitter peptides were released, resulting in a very bitter product. After further treatment with AOAP, the bitterness of this hydrolysate was significantly reduced, the umami taste was greatly enhanced, and the overall acceptability of the combined enzymatic hydrolysate was substantially improved. The total amount of free amino acids (bitter, sweet, and umami) in the combined hydrolysate reached its highest level, with bitter amino acids accounting for half. However, the TAV value of the umami amino acid glutamic acid reached 10.71, far exceeding those of the other amino acids. Combined enzymatic hydrolysis effectively eliminates most short-chain aldehydes and ketones responsible for fishy odors from the raw material, while producing long-chain aldehydes with pleasant aromatic properties. Of particular note, dibutyl phthalate was detected in all hydrolysate samples with stable content. The release of this pollutant will facilitate its subsequent removal, thereby mitigating potential risks to human health. This characteristic may offer an additional advantage of enzymatic hydrolysis over traditional fermentation, provided that an effective post-hydrolysis removal step for pollutants (e.g., dibutyl phthalate) can be practically implemented.

This study demonstrates a positive role of AOAP in improving hydrolysis efficiency and flavor under the tested conditions. These findings suggest that AOAP has potential for further optimization and application, although additional studies—including pilot-scale trials and assessment of economic feasibility—are warranted.

## Figures and Tables

**Table 1:** Types and conditions of proteases used for enzymatic hydrolysis of clam protein.

Protease Type	Optimum Temperature	Optimum pH	Enzyme Activity
Papain	50 °C	6.5	5000 U/g
Trypsin	37 °C	7.0	5000 U/g
Alkaline protease	50 °C	9.5	5000 U/g
Acid protease	50 °C	3.0	5000 U/g
Neutral protease	50 °C	7.0	5000 U/g
Flavourzyme	50 °C	7.0	5000 U/g
AOAP	50 °C	7.0	5000 U/g

**Table 2:** Scoring criteria for sensory evaluation.

Score	Aroma	Umami	Fishy Odor	Bitterness	Color	Overall Acceptability
9–10	Intense aroma	Pronounced umami	Strong fishy odor	Unbearably bitter	Dark brown	High
7–8	Strong aroma	Strong umami	Distinct fishy odor	Noticeably bitter	Brown	Relatively high
5–6	Mild aroma	Moderate umami	Moderate fishy odor	Slightly bitter	Yellowish brown	Moderate
3–4	Very faint aroma	Faint umami	Faint fishy odor	Barely perceptible bitterness	Light yellow	Relatively low
1–2	No aroma	No umami	No fishy odor	No bitterness	Colorless	Low

**Table 4:** Free amino acid contents of clam hydrolysates (mg/100 g).

Taste	Amino Acids	Control	Alkaline Protease	AOAP	Alkaline Protease + AOAP
Umami	Asp	-	34.00 ± 5.02 c	46.33 ± 3.70 b	131.00 ± 3.00 a
	Glu	78.33 ± 10.27 c	136.10 ± 3.40 b	66.50 ± 1.22 c	321.25 ± 5.25 a
	Total	78.33 ± 10.27	170.00 ± 6.06	112.83 ± 3.90	452.25 ± 6.05
Sweetness	Thr	-	58.17 ± 6.55 c	70.67 ± 2.25 b	261.75 ± 1.75 a
	Ser	-	36.33 ± 7.66 bc	48.33 ± 4.48 b	128.75 ± 3.75 a
	Gly	67.50 ± 8.60 c	142.83 ± 5.86 b	64.33 ± 3.70 c	205.25 ± 2.25 a
	Ala	-	-	-	-
	Lys	-	37.50 ± 8.83 c	85.00 ± 12.83 b	169.00 ± 4.50 a
	Pro	-	166.67 ± 2.32 b	62.50 ± 2.94 c	250.75 ± 3.25 a
	Total	67.50 ± 8.60	441.50 ± 14.81	330.83 ± 14.56	1015.50 ± 7.28
Bitterness	Val	-	48.33 ± 4.73 b	44.33 ± 3.32 b	187.00 ± 6.00 a
	Met	-	33.00 ± 6.01 b	-	105.25 ± 2.75 a
	IIe	-	63.50 ± 1.47 b	51.33 ± 4.13 bc	156.25 ± 4.25 a
	Leu	82.67 ± 4.94 d	186.33 ± 7.35 b	101.83 ± 5.54 c	363.25 ± 7.75 a
	Tyr	-	132.17 ± 3.66 b	52.83 ± 5.54 c	241.75 ± 1.25 a
	Phe	140.67 ± 7.19 c	218.83 ± 8.57 b	221.83 ± 21.83 b	292.75 ± 4.75 a
	His	-	-	-	51.25 ± 5.25 a
	Arg	-	-	-	161.00 ± 3.40 a
	Total	223.34 ± 8.72	682.16 ± 14.20	472.15 ± 23.79	1558.50 ± 13.60
Total		369.17 ± 15.98	1293.66 ± 21.39	915.81 ± 28.16	3026.25 ± 16.57

“-” indicates not detected. a–d Different letters in the same column indicate significant differences (*p* < 0.05).

**Table 5:** Taste activity values (TAVs) of free amino acids in clam hydrolysates.

Amino Acids	Control	Alkaline Protease	AOAP	Alkaline Protease + AOAP
Asp	-	0.34	0.46	1.31
Glu	2.61	4.54	2.22	10.71
Thr	-	0.22	0.27	1.01
Ser	-	0.24	0.32	0.86
Gly	0.52	1.10	0.49	1.58
Ala	-	-	-	-
Lys	-	0.75	1.70	3.38
Pro	-	0.56	0.21	0.84
Val	-	0.32	0.30	1.26
Met	-	0.44	-	1.40
IIe	-	0.71	0.57	1.74
Leu	0.44	0.98	0.54	1.91
Tyr	-	-	-	-
Phe	1.56	2.43	2.46	3.25
His	-	-	-	2.56
Arg	-	-	-	3.22

“-” indicates not detected.

**Table 6:** Volatile compounds identified in clam hydrolysates.

Classification	Compound Name	Chemical Formula	CAS No.	Control	Alkaline Protease	AOAP	Alkaline Protease + AOAP
				Content (μg/g)			
Aldehydes	2-ethylbutenal	C_6_H_10_O	19780-25-7	0.15 ± 0.04	-	-	-
	4-pyridinecarboxaldehyde	C_6_H_5_NO	872-85-5	-	-	0.04 ± 0.03	-
	hexanal	C_6_H_12_O	66-25-1	0.11 ± 0.01	-	-	-
	benzaldehyde	C_7_H_6_O	100-52-7	0.14 ± 0.04	-	0.06 ± 0.01	-
	heptanal	C_7_H_14_O	111-71-7	0.10 ± 0.03	-	-	-
	trans-2-octenal	C_8_H_14_O	2548-87-0	0.06 ± 0.01	-	-	-
	phenylacetaldehyde	C_8_H_8_O	122-78-1	-	-	0.02 ± 0.02	-
	octanal	C_8_H_16_O	124-13-0	0.12 ± 0.03	-	-	-
	trans-2-nonenal	C_9_H_16_O	18829-56-6	0.08 ± 0.01	-	-	-
	nonanal	C_9_H_18_O	124-19-6	0.08 ± 0.01	-	-	-
	decanal	C_10_H_20_O	112-31-2	0.08 ± 0.03	-	0.08 ± 0.00	0.10 ± 0.04
	trans-2-decenal	C_10_H_18_O	3913-81-3	0.17 ± 0.04	-	-	-
	2,4,5-trimethylbenzaldehyde	C_10_H_12_O	5779-72-6	0.46 ± 0.25	-	0.55 ± 0.02	0.55 ± 0.03
	syringaldehyde	C_10_H_16_O_2_	53447-47-5	-	-	0.08 ± 0.01	0.31 ± 0.03
	2-butyl-2-octenal	C_12_H_22_O	13019-16-4	-	-	0.14 ± 0.12	0.16 ± 0.02
	dodecanal	C_12_H_24_O	112-54-9	0.10 ± 0.02	-	0.08 ± 0.02	1.86 ± 0.15
	tetradecanal	C_14_H_28_O	124-25-4	0.14 ± 0.02	-	-	-
	pentadecanal	C_15_H_30_O	2765-11-9	0.11 ± 0.02	-	0.07 ± 0.01	0.09 ± 0.01
	hexadecanal	C_16_H_32_O	629-80-1	-	-	0.48 ± 0.01	0.27 ± 0.11
	heptadecanal	C_17_H_34_O	629-90-3	-	-	0.36 ± 0.11	-
	octadecanal	C_18_H_36_O	638-66-4	-	-	1.03 ± 0.08	0.37 ± 0.00
Ketones	(3E,5E)-octa-3,5-dien-2-one	C_8_H_12_O	38284-27-4	0.12 ± 0.03	-	0.09 ± 0.06	-
	octa-3,5-dien-2-one	C_8_H_12_O	30086-02-3	0.41 ± 0.16	-	0.10 ± 0.00	-
	2-nonanone	C_9_H_18_O	821-55-6	0.06 ± 0.01	-	-	-
	isopersienone	C_10_H_12_O_2_	34348-59-9	0.43 ± 0.07	-	-	-
	2-undecanone	C_11_H_22_O	112-12-9	-	0.45 ± 0.03	-	-
	2-pentylcyclohexan-1-one	C_11_H_20_O	32362-97-3	0.22 ± 0.17	-	-	0.10 ± 0.04
Furans	2,3-dihydrofuran	C_4_H_6_O	1191-99-7	0.02 ± 0.01	-	-	-
	3-acetyl-2,5-dimethylfuran	C_8_H_10_O_2_	10599-70-9	0.15 ± 0.10	-	-	-
Alkanes and Alkenes	1-undecene	C_11_H_22_	821-95-4	-	0.95 ± 0.20	-	-
	n-hexadecane	C_16_H_34_	544-76-3	0.06 ± 0.01	-	-	-
	n-heptadecane	C_17_H_36_	629-78-7	0.06 ± 0.02	-	-	-
	heptadecyloxirane	C_19_H_38_O	67860-04-2	-	-	0.21 ± 0.05	-
Alcohols	trans-3-hexen-1-ol	C_6_H_12_O	928-97-2	-	-	0.01 ± 0.01	-
	Cyclododecanemethanol	C_13_H_26_O	1892-12-2	-	-	0.08 ± 0.02	-
	cedro l	C_15_H_26_O	77-53-2	-	-	-	0.22 ± 0.02
	1,16-hexadecanediol	C_16_H_34_O_2_	7735-42-4	-	-	0.08 ± 0.01	-
Aromatic	1-(2-chloroethyl)-4-vinylbenzene	C_10_H_11_Cl	90794-48-2	-	-	0.26 ± 0.11	-
	2,4-di-tert-butylphenol	C_14_H_22_O	96-76-4	1.74 ± 0.40	0.58 ± 0.02	1.29 ± 0.51	0.10 ± 0.02
	1,3-di-tert-butylbenzene	C_14_H_22_	1014-60-4	0.68 ± 0.08	0.65 ± 0.05	0.23 ± 0.05	0.34 ± 0.01
	4-isopropoxy-N-phenylaniline	C_15_H_17_NO	101-73-5	-	-	0.75 ± 0.15	-
	galaxolide	C_18_H_26_O	1222-05-5	0.04 ± 0.01	-	-	-
Esters	3-pentyl 2,6-difluorobenzoate	C_12_H_14_F_2_O_2_	1000325-73-9	-	-	0.25 ± 0.02	-
	dibutyl phthalate	C_16_H_22_O_4_	84-74-2	-	0.30 ± 0.02	0.48 ± 0.27	0.36 ± 0.05
	2,2,4-trimethyl-1,3-pentanediol diisobutyrate	C_16_H_30_O_4_	6846-50-0	0.03 ± 0.01	-	0.07 ± 0.00	-
	diethyl 2,6-dimethyl-1,4-dihydro-3,5-pyridinedicarboxylate	C_17_H_19_NO_4_	70677-78-0	-	-	0.27 ± 0.25	-
	isopropyl myristate	C_17_H_34_O_2_	110-27-0	0.98 ± 0.54	0.18 ± 0.05	0.25 ± 0.04	0.14 ± 0.07
	ethyl palmitate	C_18_H_36_O_2_	628-97-7	0.04 ± 0.01	-	1.02 ± 0.10	-
	4-[2-[methyl(2-phenylethyl)amino]ethyl]phenyl acetate	C_19_H_23_NO_2_	52059-48-0	-	-	0.27 ± 0.06	-
	diethyl phenylethylmalonate	C_23_H_28_O_4_	76-67-5	-	-	-	0.09 ± 0.03

“-” indicates not detected.

## Data Availability

The original contributions presented in this study are included in the article/Supplementary Materials; further inquiries can be directed to the corresponding authors.

## Supplementary Materials

The following supporting information can be downloaded at: https://www.mdpi.com/article/10.3390/foods15101753/s1, Figure S1: Substrate specificity of AOAP; Figure S2: Enzymatic properties of the purified aminopeptidase (AOAP); Table S1: Effect of different concentration of protease inhibitor on AOAP activity; Table S2: Effect of different concentrations of metal ions on AOAP activity.
