# Supplementary material for: Impact of Aspergillus oryzae-Derived Aminopeptidase Complex in Developing the Flavor Profile of Clam Hydrolysate
Source: Foods. 2026 May 15;15(10):1753. doi: 10.3390/foods15101753 (PMC13206029; doi:10.3390/foods15101753)
Supplement: Supplementary file 1 [file foods-15-01753-s001.zip › foods-4292984-supplementary.pdf]

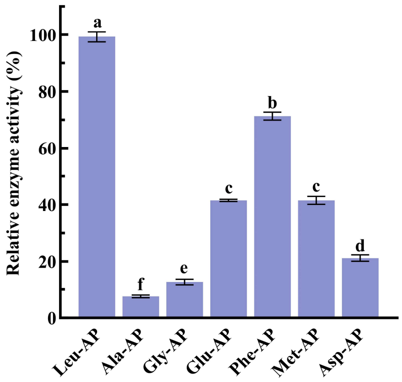

Figure S1. Substrate specificity of AOAP

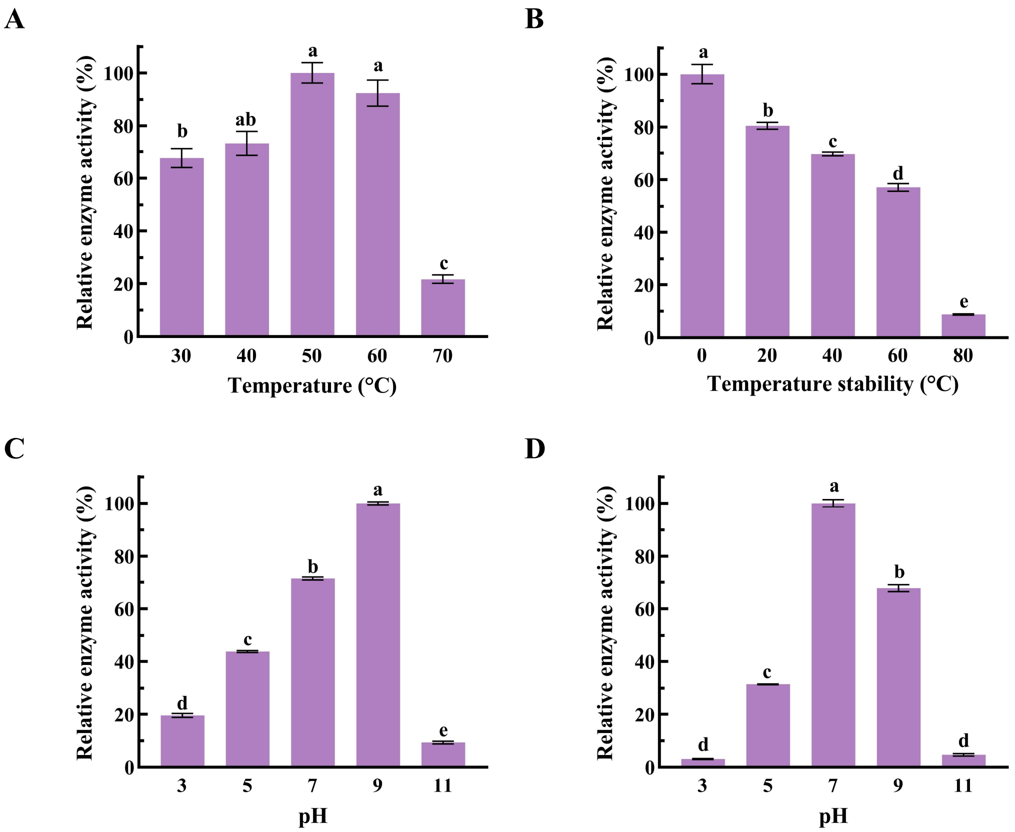

Figure S2. Enzymatic properties of the purified aminopeptidase (AOAP). (A) Optimum reaction temperature. (B) Thermal stability. (C) Optimum reaction pH. (D) pH stability.

Table S1. Effect of different concentration of protease inhibitor on AOAP activity.

Protease Inhibitor	Relative Activity (%)		
	0.01mmol/L	0.1mmol/L	1mmol/L
PMSF	84	82	97
1,10-Phenanthroline	79	42	13
E-64	81	80	94

Table S2. Effect of different concentrations of metal ions on AOAP activity.

Metal Ions	Relative Activity (%)		
	0.1mmol/L	0.5mmol/L	1mmol/L
Control	100	100	100
Mg^{2+}	108	87	52
Cu^{2+}	105	64	54
Co^{2+}	111	146	120
Zn^{2+}	95	102	87
Ba^{2+}	42	19	13
Fe^{2+}	58	92	90
